# Effect of cold stratification on seed germination in *Solidago* × *niederederi* (*Asteraceae*) and its parental species

**DOI:** 10.2478/s11756-018-0113-7

**Published:** 2018-09-03

**Authors:** Artur Pliszko, Kinga Kostrakiewicz-Gierałt

**Affiliations:** 1 Department of Taxonomy, Phytogeography and Palaeobotany, Institute of Botany, Jagiellonian University, Gronostajowa 3, 30-387 Kraków, Poland; 2 Department of Plant Ecology, Institute of Botany, Jagiellonian University, Gronostajowa 3, 30-387 Kraków, Poland

**Keywords:** Alien species, Hybrid, Seed dormancy, Timson’s index

## Abstract

In this study, we investigated the influence of cold stratification on seed germination in *S.* × *niederederi*, a hybrid between the North American *S*. *canadensis* and the European *S*. *virgaurea*, using fruit samples collected in 2016 in Poland. We aimed to test the hypothesis that the low temperature exposure decreases the final percentage and speed of seed germination in the hybrid and its parental species. For each species, sets of 100 achenes in three replications were mixed with dry sand and stored in Petri dishes in darkness for 12 weeks, at −18 °C and + 4 °C, and + 25 °C. The seeds were incubated for 21 d at room temperature (+25 °C), under the 12 h photoperiod (630 lx). We showed a lack of significant differences in: (i) the final percentage of germinated seeds of studied species stored at the same conditions, (ii) the final percentage of germinated seeds between the applied stratification conditions in the hybrid and its parental species, and (iii) the mean values of Timson’s index, mean germination time, and coefficient of velocity of germination between the stratification conditions in each species. The statistically significant inter-specific differences in the mean germination time parameter after the +25 °C treatment suggest that the seeds of *S*. × *niederederi* are able to germinate faster than the seeds of its parental species. However, to improve our knowledge of naturalization and invasion abilities of *S.* × *niederederi* by sexual reproduction, the seed germination and seedling survival of the hybrid should be tested in the field.

## Introduction

Production of viable seeds is one of the most important factors facilitating the naturalization and invasion success of alien plant species, especially when vegetative reproduction does not exist in the wild or is highly restricted (Pyšek et al. 2004; Richardson and Pyšek 2012; Bufford and Daehler 2014). Taking into account the natural hybrids between alien and native plant species, which are treated as alien species (Pyšek et al. 2004), the naturalization by sexual reproduction is usually limited by their typical low pollen viability (Daehler and Carino 2001; Stace et al. 2015). The genus *Solidago* L. (*Asteraceae*) includes many interspecific hybrids that occur in native ranges of their parental species (Nesom 1994). Nevertheless, there are two spontaneous hybrids between alien and native *Solidago* species recorded in Europe, namely *S*. × *niederederi* Khek, a hybrid between the North American *S. canadensis* L. and the European *S*. *virgaurea* L. (Nilsson 1976; Pliszko 2015; Pliszko and Zalewska-Gałosz 2016), and *S*. × *snarskisii* Gudžinskas & Žalneravičius, a hybrid between the North American *S. gigantea* Ait. and the European *S*. *virgaurea* (Gudžinskas and Žalneravičius 2016). The naturalization of both hybrids is insufficiently recognized and sexual reproduction was confirmed only in *S*. × *niederederi* (Gudžinskas and Žalneravičius 2016; Pliszko and Kostrakiewicz-Gierałt 2017a, b). Testing seed germination of hybrids between alien and native plant species under laboratory conditions is important for better recognition of their biology and may find application in developing methods of their control.

*Solidago* × *niederederi* has been reported from several countries in Europe, including Austria, Italy, the United Kingdom, Sweden, Denmark, Norway, Germany, Poland, Lithuania, Latvia, and Russia (Jaźwa et al. 2018 and literature cited therein). It is usually found among its parental species, in anthropogenic habitats such as abandoned fields, disused quarries, roadside verges, railway embankments, tree plantations, and arable fields with grass-legume mixtures (Nilsson 1976; Burton 1980; Sunding 1989; Stace et al. 2015; Gudžinskas and Žalneravičius 2016; Pliszko and Jaźwa 2017; Pliszko and Kostrakiewicz-Gierałt 2017a). It is able to spread generatively by wind-dispersed achenes; however, its fruit set is limited due to reduced pollen viability (Migdałek et al. 2014; Karpavičienė and Radušienė 2016) and depends on the abundance of mating partners (including the parental species) and pollinators (Pagitz 2016). Furthermore, *S.* × *niederederi* shows mostly self-incompatibility and therefore it can pose a threat to native *S*. *virgaurea* since its pollination biology promotes cross-hybridization and introgression (Pagitz 2016).

Interestingly, some authors suggested that *S*. *canadensis* and its closely related congeners need to receive a cold stratification to break their seed dormancy, while others pointed out that the cold temperature exposure is not required to trigger seed germination (Werner et al. 1980; Walck et al. 1997; Weber 2000). Moreover, Milbau et al. (2009) found no significant effect of cold stratification on seed germination in *S*. *virgaurea*. According to Pliszko and Kostrakiewicz-Gierałt (2017a, b), seeds of *S.* ×*niederederi* can reach a high percentage of germination (more than 90%) with no cold stratification treatment involved. However, since the hybrid is an alien species and can pose a threat to native *S*. *virgaurea* by competition and introgression, its seed germination biology should be identified in many respects. In this study, therefore, we aimed to investigate the influence of cold stratification on seed germination in *S.* × *niederederi* and its parental species by testing the hypothesis that the low-temperature exposure decreases the final percentage and speed of seed germination.

## Materials and methods

### Fruit sampling and storage

Fruit samples of *Solidago* × *niederederi*, *S*. *canadensis*, and *S*. *virgaurea* were collected from natural populations (one population per species) occurring on an abandoned field in Warsaw, central Poland (GPS coordinates: 52°06.946′N/20°59.534′E; altitude: 104 m a.s.l.), on October 2, 2016. For each species, 10 panicles (synflorescences) with mature achenes were randomly sampled, placed in paper bags, and transported to the laboratory. The panicles of the parental species were collected from the plants forming the clumps located quite far from each other (100 m), in contrast to the panicles of the hybrid which were collected from plants located close to the parental species (1–5 m). In the laboratory, the collected panicles were left in a dry, airy place, at room temperature for 7 days. Next, the panicles were threshed manually to obtain a mixture of achenes for further investigation. For each species, sets of 100 achenes in three replications were randomly selected from the samples visually identified as well-developed fruits (with no abnormalities and damage), using a PZO Warszawa 18,890 stereoscopic microscope. During an after-ripening period, sets of 100 achenes in three replications were mixed with 80 g of dry sand as a substrate and stored in 9 cm diameter polystyrene Petri dishes for 12 weeks in darkness, under three temperature regimes, namely −18 °C (in a freezer), +4 °C (in a fridge), and + 25 °C (in a room).

### Seed germination test

Achenes mixed with the sand and placed in Petri dishes (as prepared for the storage) were wetted with 10 ml of sterile water. The substrate was distributed uniformly to create a layer thickness of about 0.5 cm and its pH value was about 7.0. The achenes in Petri dishes were incubated for 21 d at room temperature (+25 °C), under 12 h photoperiod (630 lx). The substrate was complemented with 1 ml of sterile water every other day. The seed was determined as germinated when the pericarp of the achene was broken showing radicle, hypocotyl or cotyledons. During the germination test, the achenes were checked with 1 d intervals.

### Germination parameters

The speed of seed germination was estimated based on three parameters, namely the Timson’s index (Timson 1965), mean germination time (Orchard 1977) and coefficient of velocity of germination (Baskin and Baskin 2014), which are commonly used in the seed germination studies (Al-Mudaris 1998; Baskin and Baskin 2014). Formulas and descriptions of these parameters are presented in Table 1. A high value of the Timson’s index indicates a fast seed germination, a high value of the mean germination time indicates a slow seed germination, and a high value of the coefficient of velocity of germination indicates a rapid seed germination. The Timson’s index and mean germination time were calculated for a 10 d seed germination test period, whereas the coefficient of velocity of germination was calculated for a total time of seed germination test period (21 d).

**Table 1:** Details of seed germination parameters used in the study

Parameter	Formula for calculation	Description
Timson’s index	Σn	n – cumulative daily germination percentage for each day of the test
Mean germination time	Σ(n_i_ × d_i_)/N	n_i_ – number of seeds germinated at day d_i_, N – total number of seeds germinated in the test
Coefficient of velocity of germination	100(A_1_ + A_2_ + … + A_x_)/ (A_1_T_1_ + A_2_T_2_ + … + A_x_T_x_)	A_1_ + A_2_ + … + A_x_ – number of seeds germinated on the first, second and final days that seedlings appeared, T_1_, T_2_ and T_x_ – number of days between sowing and first, second and final times that seedlings were recorded

### Statistical analysis

The non-parametric Kruskal-Wallis H test with multiple comparisons was applied to check if there are significant: (i) inter-species differences in the mean percentage of germinated seeds between *Solidago* × *niederederi, S. canadensis*, and *S. virgaurea* subjected to the same fruit storage treatment, (ii) differences in the mean percentage of germinated seeds of each species between different fruit storage treatments, (iii) inter-species differences in the mean values of Timson’s index, mean germination time and coefficient of velocity of germination calculated for seeds subjected to the same fruit storage treatment, and (iv) differences in the mean values of germination parameters in each species between different fruit storage treatments. Statistical analysis was performed using a STATISTICA 13 software package.

## Results

**Fig. 1:**
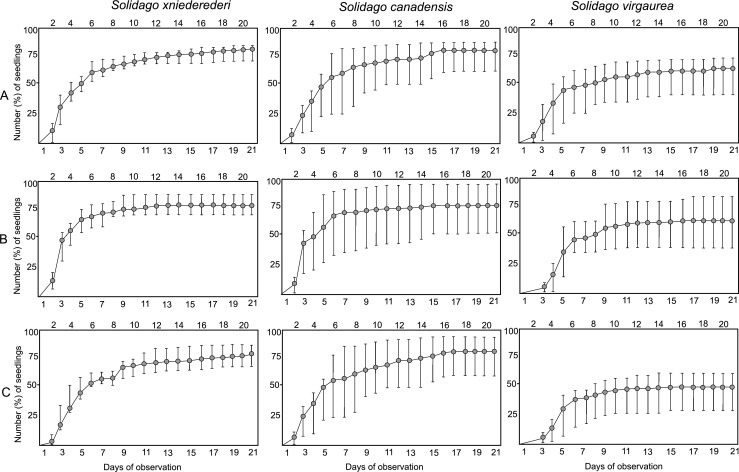
Cumulative number and percentage of germinated seeds (grey circles) and minimal and maximal values (whiskers) in *Solidago* × *niederederi* and its parental species, following −18 °C (A), +4 °C (B), and + 25 °C (C) stratification treatments, based on three replications

Seeds of *Solidago* × *niederederi* and its parental species started to germinate in the 2nd or 3rd day from the sowing and the number of germinated seeds was the greatest within the first week of the germination test period, regardless of stratification conditions (Fig. 1). The lowest values of the final percentage of germination were achieved by seeds of *S. virgaurea*, after each stratification treatment. Additionally, it should be pointed out that the seeds of *S.* × *niederederi* after the −18 °C cold stratification presented the greatest germination rate (81.3% on average). However, the inter-specific differences in the mean percentage of germinated seeds stored in the same conditions were statistically insignificant, following the Kruskal-Wallis H test (Table 2). Moreover, the differences in the mean percentage of germinated seeds between the applied fruit storage conditions were statistically insignificant in the hybrid (H = 0.8, * P = 0.6*) and its parental species (H = 0.8, *P = 0.6* for *S. canadensis* and H = 1.7, *P = 0.4* for *S. virgaurea*).

**Table 2:** The statistical significance of differences in the mean percentage (range) of germinated between *Solidago* ×*niederederi* and its parental species subjected to three stratification treatments, based on three replications

Temperature of stratification treatment	Taxon	Mean percentage of germinated seeds	The level of statistical significance
−18 °C	*Solidago* ×* niederederi*	81.3 (74–87)	3.5; *P* = 0.2
	*Solidago canadensis*	74.3 (50–94)	
	*Solidago virgaurea*	56.7 (37–69)	
+4 °C	*Solidago* × *niederederi*	76.0 (68–83)	2.3; *P* = 0.3
	*Solidago canadensis*	78.3 (57–89)	
	*Solidago virgaurea*	58.7 (37–82)	
+25 °C	*Solidago* × *niederederi*	80.7 (74–89)	3.5; *P* = 0.2
	*Solidago canadensis*	73.0 (45–88)	
	*Solidago virgaurea*	45.7 (26–56)	

The highest mean values of Timson’s index (535.0) and coefficient of velocity of germination (23.4) were noticed in *S.* × *niederederi* subjected to the +4 °C cool stratification, whereas the highest mean value of the mean germination time was noticed in *S*. *virgaurea* subjected to the −18 °C and + 25 °C storage treatments (Table 3). However, the differences in the mean values of germination parameters between the applied fruit storage conditions were statistically insignificant in each species, according to the Kruskal-Wallis H test (Table 3). On the other hand, the inter-specific differences in the mean value of Timson’s index, mean germination time, and coefficient of velocity of germination in particular types of fruit storage conditions were statistically insignificant, except the mean germination time after the +25 °C stratification treatment (Table 3).

**Table 3:** The mean (range) values of seed germination parameters in *Solidago* ×* niederederi* and its parental species after three stratification treatments

Seed germination parameter	Taxon	Temperature of stratification treatment			The level of statistical significance
		−18 °C	+4 °C	+25 °C	
Timson’s index	*Solidago* × *niederederi*	535.0 (467–600)	430.6 (401–490)	518.7 (437–617)	H = 3.31^ns^
	*Solidago canadensis*	481.7 (213–663)	406.3 (174–553)	393.3 (181–500)	H = 1.7^ns^
	*Solidago virgaurea*	341.7 (135–453)	300.0 (198–438)	241.3 (114–315)	H = 0.8^ns^
	The level of statistical significance	H = 3.3^ns^	H = 1.7^ns^	H = 4.4^ns^	
Mean germination time	*Solidago* ×* niederederi*	4.0 (3.4–4.5)	4.3 (3.8–4.7)	4.2 (4.0–4.3)^a^	H = 0.6^ns^
	*Solidago canadensis*	3.9 (3.6–4.5)	5.2 (4.4–6.5)	5.3 (4.9–5.8)^b^	H = 4.6^ns^
	*Solidago virgaurea*	4.7 (4.1–5.7)	5.6 (5.2–6.0)	5.6 (5.0–6.2)^b^	H = 1.7^ns^
	The level of statistical significance	H = 3.5^ns^	H = 1.4^ns^	H = 5.7*	
Coefficient of velocity of germination	*Solidago* × *niederederi*	23.4 (16.6–28.0)	18.1 (14.7–20.8)	21.4 (19.0–24.1)	H = 2.5^ns^
	*Solidago canadensis*	21.1 (12.6–25.6)	16.3 (11.0–20.3)	17.2 (13.5–19.2)	H = 1.1^ns^
	*Solidago virgaurea*	21.5 (14.5–28.1)	16.7 (15.4–17.7)	16.8 (14.5–18.5)	H = 0.9^ns^
	The level of statistical significance	H = 0.7^ns^	H = 0.4^ns^	H = 4.6^ns^	

## Discussion

Considering the results obtained in this study, we must reject our hypothesis that the cold stratification decreases the final percentage and speed of germination in *Solidago* × *niederederi* and its parental species. Nonetheless, a lack of influence of cold stratification on final percentage of seed germination in *S*. *canandensis* corresponds with the findings provided by Werner et al. (1980). Moreover, the outcomes of the performed studies support the observations made by Milbau et al. (2009) who noticed that the cold stratification has no effect on the final percentage of germinated seeds in *S*. *virgaurea.* Additionally, Bochenek et al. (2016) showed that the high seed vigor in *S. gigantea* Ait., a species closely related to *S. canadensis*, was maintained after the storage in a wide range of temperatures, in both dry and moist conditions. In light of aforementioned studies, it might be stated that the species, whose seeds germinate easily (regardless of storage conditions), possess the competitive advantage over the species requiring specific conditions to break their seed dormancy. Such ability seems to be an attribute of *S*. × *niederederi* and may facilitate its naturalization by sexual reproduction in new areas. On the other hand, it should be mentioned that there are several studies proving that the cold stratification breaks dormancy and improves the seed germination in *S. altissima* L., *S. nemoralis* Ait. (Walck et al. 1997, 2000), *S. petiolaris* Ait. (Bratcher et al. 1993), *S. sempervirens* L. (Lonard et al. 2015) and *S. shortii* Torr. & Gray (Buchele et al. 1991; Walck et al. 1997, 2000). Furthermore, the importance of cold stratification was confirmed in many species of the Asteraceae. For example, the cold stratification breaks seed dormancy in *Echinacea angustifolia* DC. (Baskin et al. 1992), *Polymnia canadensis* L. (Bender et al. 2003), *Guizotia scabra* (Vis.) Chiov., *Parthenium hysterophorus* L.*, Verbesina encelioides* (Cav.) Benth. & Hook. f. ex A. Gray (Karlsson et al. 2008), as well as *Tripleurospermum maritimum* (L.) W. D. J. Koch (Bochenek et al. 2010), while a moderate thermal stratification has a positive influence on seed germination in *Cirsium arvense* (L.) Scop. (Bochenek et al. 2009).

Interestingly, our results suggest that *S*. ×*niederederi* can reach much higher values of the final percentage of seed germination than evidenced by Pagitz (2016) and correspond with the previously published data (Pliszko and Kostrakiewicz-Gierałt 2017a, b). Moreover, the final percentage of germinated seeds in *S. canadensis* was also higher than evidenced by other authors (Huang et al. 2007) and a low final percentage of seed germination in *S. virgaurea* corresponds with the results provided by Giménez-Benavides et al. (2005). At the same time, it should be pointed out that the high percentage of germinated seeds may not result in a considerable abundance of adult individuals. Goldberg and Werner (1983) showed that in the closely related *S. altissima* the seedling growth and their probability of survival increased with the diameter of the opening in the vegetation. Based on laboratory observations, Hou et al. (2014) evidenced the considerable mortality of seedlings in many invasive species from the Asteraceae family (e.g., *Eupatorium catarium* Veldkamp, *Ageratum conyzoides* L., *Tridax procumbens* L., *Mikania micrantha* Kunth, and *Synedrella nodiflora* (L.) Gaertn.) in effect of low temperatures. Findings of numerous authors showed that the unfavorable weather conditions may lead to a loss of seedlings in the wild. For example, the sowing experiments conducted by Poll et al. (2008) in the field showed the slight survival of seedlings of *S. canadensis*, *Conyza canadensis* (L.) Cronquist, and *Matricaria discoidea* DC. Furthermore, the suppression of seedling establishment in effect of severe drought or strong late frost was noticed in invasive *Fallopia japonica* (Houtt.) Ronse Decr. (Engler et al. 2011; Funkenberg et al. 2012; Forman and Kesseli 2003).

In comparison to Walck et al. (1997), who evidenced that in *S. altissima*, *S*. *nemoralis*, and *S. shortii*, the effect of stratification treatment on Timson’s index was species-specific, we found no significant difference in the Timson’s index value between *S*. × *niederederi* and its parental species (Table 3). However, regarding the statistically significant inter-specific differences in the mean germination time parameter after the +25 °C treatment (Table 3), it should be stated that the seeds of *S*. × *niederederi* are able to germinate faster than the seeds of its parental species. On the other hand, similarly to *S. altissima*, *S*. *nemoralis*, and *S. shortii* (Walck et al. 1997), the greatest number of germinated seeds in the hybrid and its parental species was noticed in the first week of incubation (Fig. 1). To be more critical of our results, we realize that in the wild, the final percentage of germinated seeds and speed of seed germination in *S*. × *niederederi* may be very different from those observed in the laboratory, therefore, it should be tested in the field as pointed out by Gioria and Pyšek (2017). Finally, many other aspects of seed ecology of the hybrid such as tolerance to drought, persistence in the soil seed bank, and interactions with the soil microorganisms seem to be interesting topics for further investigation on its naturalization and invasion abilities.
